# The Opium Curse

**Published:** 1893-01

**Authors:** 


### THE OPIUM CURSE.

It is surprising that this government has allowed one of the most debasing habits that ever cursed the human race to obtain a footing in this land, and more than all, that the baneful evil has been suffered to spread from city to city, wherever its semi-barbarian conspirators against the good health and morals of a people have obtained a lodgment. It is only now and then that, with some show of bluster, an official raid is made upon one of those dens of iniquity known as an "opium joint," and whenever this occurs, more or less young American girls are found among the besotted slaves of vice. It is no bar to the indulgence of this wickedness that it is against the law. The law is too indulgent. A mere fine is not enough. Confiscation and banishment would be a more adequate penalty for this willful corrupting of body and soul of weak young men and innocent young girls. The following dramatic picture, which we find in the editorial columns of the *Pacific Medical Journal*, is as common of New York as of San Francisco :

"Some time ago we were called to see a young lady who was supposed to be dying. We were conducted along a small narrow street leading off Dupont street near Jackson. On arriving at the destination we were shown into a room about twenty feet square, well guarded by outside and inside sentinels. On the matted floor and cushioned divans lay fourteen men and women, all busy with long clumsy looking pipes, small steel rods and tiny boxes containing a tarry looking material. They were diligently 'cooking' small pellets of this material —opium—over small lamps, placing them in their pipes, lighting them and eagerly smoking the drug. A veritable 'opium den.' Of these fourteen people some were Chinese, but most of them were white girls and young white men. Here they lay, side by side, men and women, of them smoking the noxious drug.
Chinese and white, in all conditions of nudity, some asleep, but most

At one end of the room lay the object of our visit, a beautiful white girl of about twenty. She was in a profound opium stupor, with pinpoint pupils and respirations eight per minute. It was learned that this was the first time she had 'hit the pipe,' and she had evidently 'hit' it pretty hard, for we worked with her most of the night before she recovered.

Many similar dens flourish in Chinatown and other parts of the city, as they do in Chicago, New York, and wherever Chinese congregate. It is high time our government took decisive steps to abolish this infamous traffic, and give our city officials a chance to close up these iniquitous opium dens, and wipe out one of the darkest spots on our fair city's escutcheon."

Something should be done to abolish this infamous traffic, and done at once. Should all plans fail, then abolish the wretches themselves who insist upon keeping it alive within our borders. No punishment could be too severe for them.

